# Factors Influencing Food Safety Education Practices among Obstetricians

**DOI:** 10.3390/healthcare11010111

**Published:** 2022-12-30

**Authors:** Hala Ayman Alyousef, Xiyu Cao, Nianhong Yang

**Affiliations:** 1 Department of Nutrition and Food Hygiene, Hubei Key Laboratory of Food Nutrition and Safety, MOE Key Laboratory of Environment and Health, School of Public Health, Tongji Medical College, Huazhong University of Science and Technology, 13 Hangkong Road, Wuhan 430030, China; 2 Department of Nutrition, Health Science Faculty, Al-Baath University, Homs 96331, Syria

**Keywords:** food safety education, obstetricians, pregnant women, barriers, attitude

## Abstract

Healthcare providers (HCPs) are responsible for educating pregnant women about food safety issues in to prevent infectious diseases; however, few HCPs provide their pregnant women with such information. This study aimed to identify food safety education practices, attitudes and barriers among obstetricians and determine the factors affecting education practices. In this cross-sectional study, 238 obstetricians completed a self-administered questionnaire. Medians with interquartile ranges, frequency, crosstabs test, Mann–Whitney U test, Kruska–Wallis H test, Dunn–Bonferroni post hoc method, and multiple regression were used for data analysis. The study found that obstetricians provide pregnant women with a low amount of food safety information (2.5 ± 0.42, and the top three barriers reported were lack of time (Mdn = 3, IQR = 1), lack of knowledge (Mdn = 3, IQR = 2), and heavy workload (Mdn = 3, IQR = 2). Furthermore, only a few obstetricians were interested in taking food safety education courses (30.2%) and exploring pregnant women’s education needs (39.5%). Factors influencing education practices were total experience, lack of knowledge, and lack of time. Obstetricians should be more aware of the need to educate pregnant women about food safety issues. Understanding the influencing factors determined in this study will contribute to the development of an effective education plan to reinforce doctors’ food safety education competency.

## 1. Introduction

A weakened cellular immune system, caused by hormonal changes during pregnancy, places pregnant women at a higher risk of foodborne diseases (FBDs) [1]. Foodborne pathogens cause severe illnesses in mothers and fetuses, including stillbirth, premature labor, and miscarriage [2,3]. These life-threatening pathogens can be avoided by adhering to simple preventive measures during pregnancy [4]. Previous studies have found that promoting consumers’ food safety practices is an important factor in preventing FBD [5,6]. These studies reported that food safety knowledge and attitudes are the most important cognitive factors influencing food safety practices [7,8,9]. Unfortunately, pregnant women are generally unaware of the severe consequences of infectious diseases during pregnancy and lack extensive food safety knowledge [10,11,12]. In addition, pregnant women often do not follow safe food handling practices and continue to consume high-risk foods during pregnancy, despite the increased risk of FBDs [10,11,12]. Therefore, several studies have highlighted the urgent need to provide pregnant women with food safety education [11,13]. Providing pregnant women with food safety education, including information on FBDs, avoiding high-risk foods, and following preventive methods, is important to increase pregnant women’s awareness and prevent infectious diseases. The source of information plays an important role in consumers’ behavioral changes, as consumers are more likely to adhere to recommendations when they are confident in the source of information [14]. Pregnant women consider their healthcare providers (HCPs) to be trusted sources of information on food safety [15,16,17,18]. In Syria, obstetricians are the most important HCPs of the maternity care team. They are the most common denominator providing care for pregnant women during pregnancy, labor, delivery, and the postnatal period. Other HCPs, such as registered nurses, midwives, or dietitians, may or may not come into contact with pregnant women. Pregnant women in Syria can choose private physicians’ clinics or public hospitals that provide free services [19]. In 2019, the maternal care system in Syria has shown considerable progress in providing health services [20]. The Damascus Maternity Teaching Hospital in Damascus and Maternity Children Hospital in Latakia are public hospitals providing free maternity services. The Damascus Maternity Teaching Hospital is the largest maternity teaching hospital in the country, with an average of 11,000–13,000 deliveries per year [21].

A previous study reported that HCPs are responsible for educating women about food safety issues; however, few HCPs were found to provide their pregnant women with such information [22,23,24,25]. Pereboom found that Dutch midwives provide pregnant women with insufficient information on infectious disease prevention [23]. In another study, 69.7% of Canadian HCPs who provided prenatal care counseled pregnant women about listeriosis. In British Columbia, most prenatal care providers were found to be not counseling pregnant women about listeriosis. Another study found that less than half of HCPs in China and Peru provided food safety information to their patients [24].

Surprisingly, few studies addressed the counseling practices of HCPs regarding informing pregnant women about food safety during pregnancy, and they were found to have a limited understanding of barriers which prevented them from providing such information. Moreover, no studies addressed the attitudes of HCPs regarding providing food safety information for pregnant women with less awareness of food safety.

Therefore, the current study’s main aim was to determine factors influencing obstetricians’ practices regarding providing food safety information to pregnant women. The specific aims were as follows:

- Identify obstetricians’ practices in providing food safety information regarding foodborne pathogens, prevention methods, and high-risk food intake.
- Identify barriers to providing food safety information to pregnant women.
- Assess obstetricians’ attitudes when counseling pregnant women about food safety.
- Assess the correlation between the selected sociodemographic factors of obstetricians and their practices in providing food safety information.
- Determine the factors influencing obstetricians’ practices regarding providing food safety information to pregnant women.

## 2. Materials and Methods

### 2.1. Study Population

The target population included doctors who provided antenatal care to pregnant women living in Syria and worked either in public hospitals and health centers that provide free maternity services or in private clinics. Doctors who did not provide antenatal care to pregnant women were excluded. To increase the coverage of the research scope across multiple regions of the Syrian Arab Republic, this study was conducted in the capital, coastal, and central regions of the country.

The minimum sample size was calculated using a sample size calculation program (G-Power software, version 3.1.9.2). A total of 184 participants were required for a moderate effect size of 0.15, power of 0.95, and alpha level of 0.05 using multiple linear regression with 12 predictors. Through a convenience sampling method, 300 obstetricians were invited to participate in this study, considering the potential dropout rate. After excluding 62 responses due to incomplete or inappropriate answers, 238 were included in the final analysis, with a response rate of 79.3%. 

### 2.2. Study Design

The present study used a descriptive cross-sectional design.

### 2.3. Methods

The authors developed a self-administered questionnaire based on related literature to identify obstetricians’ practices, barriers, and attitudes regarding providing food safety education to pregnant women [22,23,24]. The questionnaire included four sections: 

(1) Sociodemographic characteristics section aimed to gather information about the general characteristics of participants (age, gender, and working experience) using multi-choice questions. 

(2) The counseling practices section included 20 items related to foodborne pathogens, preventive practices, and high-risk foods for pregnant women, with responses rated on 5-point Likert scale, ranging from 1 = “never” to 5 = “always”. The total scores of this section ranged from 20 to 100; the higher the total score, the higher the rate of food safety counseling.

(3) The barriers section (eight items) aimed to identify barriers preventing counseling practices, with question responses recorded using a 4-point Likert scale, varying from 1 = “strongly disagree” to 4 = “strongly agree”. The total score ranged from 8 to 32; the higher the total score, the more significant the food safety education barriers.

(4) The attitude section (six items) aimed to explore the obstetricians’ attitudes toward pregnant women groups, with responses recorded using a 4-point Likert scale, varying from 1 = “strongly disagree” to 4 = “strongly agree”. The total score for this section ranged from 6 to 24; the higher the score, the more positive the attitude.

The study was conducted from February to June 2021. A hard copy questionnaire was distributed to participants and collected on the spot after they were completed. Informed consent was obtained, and no incentives were provided for participation. An information sheet explaining the study’s aims was presented on the first page of the questionnaires. The research assistants were present to answer any questions that the respondents might have had. On average, the participants needed 10 min to complete the questionnaire. The questionnaire was written in English and then translated into Arabic. Five relevant senior lecturers at Al Baath University in Syria evaluated the questionnaire for content validity, and minor revisions were made to the wording of the questions based on the feedback received. After that, the questionnaire was pilot tested by 15 obstetricians (not included in the actual study) to test the questionnaire’s reliability, clarity, adequacy, and effectiveness. The Cronbach’s alpha for the scale was 0.846. Analyzing the scale reliability based on the scale sections, Cronbach’s alpha was 0.823 for the practice scale, 0.721 for the barrier scale, and 0.719 for the attitude scale. The survey was determined to be acceptable and understandable. The study was approved by Al Baath University in Homs (No: 192-S) and got permission from Damascus Maternity Teaching Hospital in Damascus (No: 7999-S) and the Maternity Children Hospital in Latakia (No: 25-H-S-623).

### 2.4. Statistical Analysis

For data analysis, IBM SPSS Statistics (version 23.0) was used. Medians with interquartile ranges and frequency were used to analyze obstetricians’ characteristics, practices, barriers, and attitudes regarding food safety education. After that, non-parametric tests, including the Mann–Whitney U test, Kruskal–Wallis H test and Dunn–Bonferroni post hoc method, were used to compare the mean rank scores of attitude, total food safety education practices, high-risk food education subscale, foodborne pathogens education subscale, and prevention practices education subscale for participants belonging to different sociodemographic groups. To explore the correlation between knowledge and other variables, crosstabs and Chi-Square tests were used. A standard multiple regression test was used to analyze the factors influencing food safety education practices. The results were considered statistically significant if the *p*-value < 0.05.

## 3. Results

A total of 300 obstetricians were invited to participate in this study. After excluding 62 responses due to incomplete or inappropriate answers, 238 were included in the final analysis, with a response rate of 79.3%. The a posteriori power was 99%. This study found that 122 (51.3%) respondents were male, and 116 (48.7%) were female. About 65% of participants were aged less than 40 years. Half of the doctors in this study had provided antenatal care to pregnant women for 9 years or less. Among the doctors who participated in this study, 35.7% provided antenatal care in the capital region, while 29.8% worked in the coastal region. Table 1 shows obstetricians’ demographic characteristics.

The overall mean score of food safety education practices was 2.5 ± 0.42 (Table 2). Regarding the foodborne pathogen subscale, the results showed that the majority of respondents sometimes provided pregnant women with information about listeria (Mdn = 3, IQR = 1) and toxoplasma (Mdn = 3, IQR = 2). However, obstetricians reported a low likelihood of educating women regarding methods to prevent FBDs subscale (2.39 ± 0.47). These results showed that the majority of obstetricians rarely informed pregnant women about hygienic practices. Regarding the high-risk food subscale, the majority of participants sometimes informed pregnant women to avoid consuming raw meat or undercooked meat (Mdn = 2, IQR = 1), as well as to avoid consuming unpasteurized dairy products (Mdn = 2, IQR = 1) and ready-to-eat meat without ensuring it is hot (Mdn = 2, IQR = 1) (supplementary data).

Age and total working experience were significantly associated with the foodborne pathogens education subscale and preventive methods (Table 3). However, Dunn–Bonferroni post hoc method showed that doctors aged 25–29 years provided significantly less information than those who were 40–49 and ≥50 years old. This study also found that doctors with 0–9 years of experience had significantly lower mean rank scores than doctors with 10–19 years of experience. By examining the education practices based on high-risk food subscales, the Dunn–Bonferroni post hoc method showed that doctors aged 25–29 years had the lowest mean rank score compared to other groups. In comparison, doctors who had worked for ≥20 years scored a significantly higher mean rank score than those who had worked for 0–9 or 10–19 years. The results also showed that gender was not significantly associated with food safety education practices subscales.

Regarding barriers to educating pregnant women about food safety issues, the total mean score was 2.7 ± 0.43, and the most commonly reported reasons were lack of time (85.8% of doctors reported agreement with this barrier (Mdn = 3, IQR = 1)), followed by lack of knowledge (76.0% of doctors reported “strongly agree” or “agree” (Mdn = 3, IQR = 2)) and heavy workload (72.6% of doctors reported agreement with this barrier (Mdn = 3, IQR = 2)) *(*Table 4). This study showed that a lack of knowledge was significantly associated with food safety education practices. Doctors who claimed to lack knowledge had significantly lower mean scores (2.39) than those who reported having knowledge (3.09) (Table 3). 

**Figure 1:**
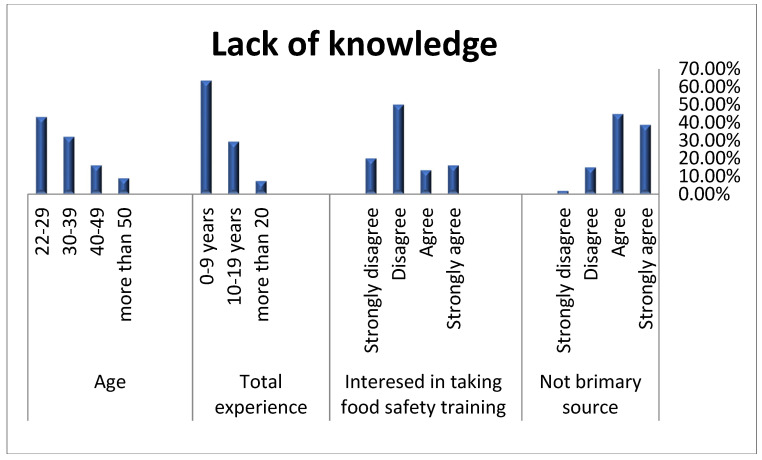
Correlation between lack of knowledge and selected variables. “Agree” and “strongly agree” were considered to be a Yes = 2 (having a lack of knowledge), while “strongly disagree” and “disagree” were considered to be a No = 1.

The chi-squared test results showed that age and years of experience were significantly related to doctors’ food safety knowledge. Only 7.2% of doctors with more than 50 years of experience reported a lack of knowledge, while 29.3% and 63.5% of doctors with 10–19 and 0–9 years of experience reported a lack of knowledge. Furthermore, the number of doctors who reported a lack of knowledge significantly decreased with the increase in their ages (Figure 1). The results also showed that more than 83% of doctors who claimed a lack of knowledge thought they were not the primary source of food safety information. Moreover, most doctors who claimed to lack knowledge were not interested in participating in food safety education programs (70.9%).

The overall mean score for food safety education attitude was 2.62 ± 0.32. Most obstetricians agreed or strongly agreed with the statements that less-educated pregnant women need more food safety counseling (78.2%) and that younger pregnant women are less aware of food safety issues (92.0%). A few obstetricians were interested in taking food safety education courses (30.2%) and exploring pregnant women’s education needs (39.5%) (Table 2).

This study found that knowledge was significantly associated with the attitudes toward the food safety education score (Table 4). Doctors who reported a lack of knowledge had lower attitude mean rank scores (108.19) compared to another group. The results also showed a significant association between age, total working experience, and attitude score (Table 4). The Dunn–Bonferroni post hoc method showed that doctors who had worked for 0–9 years obtained lower attitude mean rank scores than those who worked for more than 20 years and those with 40–49 years of work experience. The results also showed that doctors over 50 years old obtained a higher mean rank score than those aged 22–29 years and 30–39 years. No significant associations were found between gender and attitude scores.

Testing the basic assumptions for multiple linear regression analysis showed that no multicollinearity in the multiple linear regression models was detected, with a < 3 variance inflation factor for all variables. The residuals of the regression are normally distributed and homoscedastic. For linear regression analysis, the responses to the barriers questions were classified as categorical variables (there is a barrier or no barrier). Multiple linear regression analysis showed that attitude (*p* = 0.001), total experience (*p* < 0.001), lack of knowledge (*p* < 0.001), and lack of time (*p* < 0.001) were significant predictors of food safety education practices (Table 5). These variables accounted for 59.9% of the variance in the final model. 

## 4. Discussion

Providing pregnant women with information about behaviors and lifestyle habits that can prevent infectious diseases is important, especially given the severity of the consequences of FBDs caused by certain pathogens. However, the study found that obstetricians provide pregnant women with a low amount of food safety information. Obstetricians reported the lowest prevalence of education practices for pregnant women regarding the prevention practices subscale, and the majority of participants rarely advised pregnant women about hygienic practices.

Furthermore, a low rate of providing pregnant women with information regarding avoiding high-risk foods was also reported. This low rate of educating women about food safety issues is in line with previous studies [22,23,24,25] and indirectly comparable with studies that found gaps in awareness regarding food safety practices and the consumption of high-risk foods among pregnant women [10,11,12]. Previous studies reported that pregnant women need more information about foodborne pathogens, including listeria, toxoplasma, and other pathogens of concern [26,27,28]; the incidence of toxoplasmosis was significantly decreased after educating pregnant women about this pathogen [29]. One study found that FBD training increased clinicians’ awareness of FBDs [30]; therefore, this study suggests further training programs for obstetricians that focus on areas where food safety education practices are insufficient. Age and extent of doctors’ experience were significantly associated with total food safety education practices and the foodborne pathogens, high-risk food, and preventive methods education subscales. Obstetricians who had worked for ≥20 years scored a significantly higher mean rank score on total food safety education practices compared to others. Likewise, young doctors were less likely to provide food safety information. It is reasonable that experienced and older doctors were more comfortable educating pregnant women about food safety issues. Lack of time was the most common barrier reported, and a possible explanation may be that doctors often provide care to a larger number of pregnant women than recommended. Clearly, this can affect the time given to each woman and result in a lower level of information about food safety issues being shared. The findings of our study are in line with another study conducted in China and Peru, in which 63% of the respondents reported a lack of time as being among the top three hindrances [24]. Furthermore, it was found that food safety education was considered a supplemental activity that required extra time and staff [24].

Most obstetricians reported a lack of knowledge as a barrier to counseling pregnant women about food safety information. Lack of knowledge was also significantly associated with food safety education practices, which could be attributed to the fact that having factual knowledge (knowing what to teach) is a significant aspect of patient education [31]. Therefore, the need to provide HCPs with more training and skills to educate pregnant women on food safety information should be emphasized. Lack of knowledge was also among the main reasons for not educating pregnant women in previous studies [15,22,32,33].

In our study, less than half of doctors reported a negative attitude toward the importance of identifying pregnant women’s education needs, which may result in less effective women’s education. To provide effective education, patient education needs must be explored [34,35,36]. The obstetricians in this study thought that women with many children needed less food safety education than women who were experiencing their first pregnancy. A possible explanation could be that doctors expect women with previous pregnancies to be more familiar with food safety issues and less receptive to such information than women who are pregnant for the first time. This assumption is reasonable; previous studies have shown that women who are pregnant with their first child were less knowledgeable and more prone to engaging in unsafe food practices compared to women who had more children [27,37]. In contrast, a previous study found that women with three or more pregnancies were less knowledgeable about listeriosis [13]. Therefore, the present study recommends that obstetricians provide the same food safety information to women who are pregnant for the first time and women who have had multiple pregnancies.

This study aimed to identify the factors influencing obstetricians’ practices regarding the provision of food safety information to pregnant women. Multiple linear regression analysis found that doctors’ total work experience was a significant factor in pregnant women’s education. This result might be because experienced doctors who have worked for a long time have improved their competence and accumulated experience in women’s education about food safety issues and risk factors. We also found that years of experience were significantly related to the doctors’ food safety knowledge, as the number of obstetricians who reported having enough knowledge increased with work experience. Innovative methods of continuing education targeting newly graduated obstetricians should be used to address identified gaps and overcome a lack of food safety knowledge. This will allow obstetricians to improve their educational competency. 

The results indicated that the lack of knowledge was the strongest factor influencing the practices of women education relating to food safety. This finding suggests that there is a real need for obstetricians to be provided with more food safety education, including updated knowledge and skills. However, the study showed that most obstetricians who claimed a lack of knowledge thought they were not the primary source of food safety information. These results represent big challenges, as most obstetricians were not interested in taking food safety education. Therefore, initiating an educational campaign intended for obstetricians is required to improve their knowledge and motivate them to provide more food safety information to pregnant women. Furthermore, the organization’s cooperation and management, practitioner, and effective communication are required to develop a successful food safety culture [38], which would motivate HCPs to educate pregnant women about food safety issues.

Lack of time was also a significant factor influencing food safety education. Time is critical to any educational process, and doctors will need to spend more time educating pregnant women. To address this barrier, delegating food safety education tasks to other medical disciplines, such as nutritionists, could alleviate time limitations, and educational tools could be used. A previous study found that HCPs in China and Peru identified videos in the waiting room as the most effective format for providing food safety education [24]. E-learning, a new educational technique, could be a great replacement for face-to-face education [39,40]. Further studies should be conducted to evaluate the effectiveness of less time-consuming education methods to provide a guide for food safety education development for HCPs and pregnant women.

Healthcare improvement is not an easy task and is usually performed at the systemic level, as it requires several organized efforts. Improving healthcare should start with developing faculty expertise to educate the next generation of HCPs about the importance of food safety and the risks of FBDs during pregnancy. One study found that the patient education performance of health physician students was effectively improved after patient education training [41]. The management of healthcare systems should create a strong food safety culture that affects all individuals and makes food safety a shared responsibility. 

The present study has some limitations. First, because this study surveyed obstetricians in Syria through a cross-sectional survey, the results cannot be generalized to other HCPs in other countries. Second, because the data for this study were collected via a self-report questionnaire, the responses may be subject to bias and may not reflect the actual behaviors of the respondents. Third, because the data were collected through the convenience sampling technique, the results cannot be generalized. Lastly, this study did not assess obstetricians’ knowledge of food safety practices.

## 5. Conclusions

During pregnancy, doctor-led food safety education is important to foster health behavior changes to protect pregnant women from FBDs. To our knowledge, this is the first evaluation of obstetricians’ food safety education performance in Syria. The present study indicated that information on food safety issues provided to pregnant women by obstetricians was insufficient, especially information on preventive practices. Attitude, total experience, lack of knowledge, and lack of time were factors associated with food safety education practices. As obstetricians are the most important HCPs of the maternity care team, and they are the most common denominator providing care for pregnant women in Syria, they need to be more aware of the need to inform pregnant women about food safety issues. Providing appropriate and tailored food safety training programs and enhancing the importance of food safety can potentially encourage obstetricians to provide food safety information. Institutional efforts are required to increase obstetricians’ awareness of the need for food safety education, along with organizational support, such as creating a positive food safety culture and providing less time-consuming education methods. Further studies should be conducted to assess the effectiveness of food safety education programs, considering the barriers and influencing factors reported in this study.

## Figures and Tables

**Table 1:** Obstetricians’ demographic characteristics.

Variable	Category	n (%)
Age		
	25–29	83 (34.9)
	30–39	73 (30.7)
	40–49	49 (20.6)
	≥50	33 (13.9)
Gender		
	Male	122 (51.3)
	Female	116 (48.7)
Total working experience (year)		
	0–9 years	128 (53.8)
	10–19 years	83 (34.9)
	≥20 years	27 (11.3)
The majority of your practice is in		
	Capital region	85 (35.7)
	Coastal region	71 (29.8)
	Central region	82 (34.5)

**Table 2:** Obstetricians’ practices, attitude, and barriers mean score regarding food safety education.

Variables (No. of Items)	Mean	Std. Deviation
Foodborne pathogen education practices (4)	2.7	0.59
Prevention practices education practices (9)	2.39	0.47
High-risk food education practices (7)	2.65	0.58
Total food safety education practices (20)	2.5	0.42
Barriers to food safety education (8)	2.75	0.43
Obstetricians’ attitudes toward food safety education (6)	2.62	0.32

**Table 3:** Association between obstetrician’s attitudes, food safety education performance, and selected variables.

Variable	Foodborne Pathogens Education Mean Rank	Prevention Methods Education Mean Rank	High-Risk Food Education Mean Rank	Total Food Safety Education Practices Mean Rank	Attitude Mean Rank
Age					
25–29	102.52	86.76	78.53	76.86	99.75
30–39	112.63	112.97	113.77	113.87	116.90
40–49	143.55	145.16	147.56	153.34	131.19
≥50	141.70	178.18	193.55	188.95	157.58
*p*-value	0.001	˂0.001	˂0.001	˂0.001	˂0.001
Gender					
Male	120.95	118.14	120.21	119.89	125.48
Female	117.97	120.94	118.75	119.09	113.22
*p*-value	0.73	0.75	0.86	0.92	0.16
Total working experience (year)					
0–9	106.61	2.19	81.29	82.16	101.21
10–19	135.02	2.55	151.74	152.63	132.71
≥20	132.93	2.90	201.52	194.69	165.59
*p*-value	0.007	˂0.001	˂0.001	˂0.001	˂0.001
Lack of knowledge					
No	194.41	187.39	181.46	202.74	155.41
Yes	95.91	98.12	99.99	93.29	108.19
*p*-value	˂alue9	˂alue9	˂alue9	˂0.001	˂0.001

Note: Statistically significant (*p* < 0.05).

**Table 4:** Obstetricians’ attitude and barriers regarding food safety education.

Variable	Strongly Disagree (%)	Disagree (%)	Agree (%)	Strongly Agree (%)	Median	Interquartile Range
Barriers to food safety education						
I do not have enough time	0.8	13.4	59.7	26.1	3.00	1.00
I do not have enough knowledge	4.2	19.7	50.8	25.2	3.00	1.00
I am not a primary source of food safety information	5.0	26.5	38.2	30.3	3.00	2.00
Pregnant women will not eat high-risk foods anyway	8.8	44.1	33.6	13.4	2.00	1.00
Heavy workload and too many patients at the clinics	2.5	24.8	55	17.6	3.00	1.00
Food safety issues are less important to discuss with pregnant women than other topics	13.9	21.8	56.3	8.0	3.00	1.00
I forget, or I need reminders	8.4	57.6	29.0	5.0	2.00	1.00
Lack of resources and suitable educational tools	5.5	29.0	55.5	10.1	3.00	1.00
Obstetricians’ attitude toward food safety education						
Less-educated pregnant women need more food safety counseling	2.1	19.7	68.1	10.1	3.00	0.00
Younger pregnant women are less aware of food safety issues	0.4	7.6	75.2	16.8	3.00	0.00
It is important to identify food safety education needs for pregnant women	1.7	58.8	35.7	3.8	2.00	1.00
Providing pregnant women with more food safety education will increase their awareness	0.8	34.5	54.6	10.1	3.00	1.00
Pregnant women who have more children need more food safety education	3.4	52.1	42.9	1.7	2.00	3.00
Are you interested in taking a continuing food safety education course?	19.7	50.0	16.8	13.4	2.00	1.00

**Table 5:** Factors influencing food safety education practices.

Variable 1	B	*p*
Constant	58.92	<0.001
Gender	−0.023	0.97
Age	0.89	0.71
Total experience	2.88	<0.001
Lack of time	−5.91	<0.001
Lack of knowledge	−8.98	<0.001
Doctors are not a primary source of food safety information	−1.64	<0.07
The pregnant women will not eat the high-risk foods anyway	0.67	0.36
I forget, or I need reminders	−0.136	0.84
Food safety issues are less important to discuss with pregnant women than other topics	0.98	0.146
Heavy work	0.155	0.85
Lack of resource	1.25	0.088
Attitude	2.32	0.036

Note: Statistically significant (*p* < 0.05).

## Data Availability

All raw data supporting reported results is available from authors upon reasonable request.

## Supplementary Materials

The following supporting information can be downloaded at: https://www.mdpi.com/article/10.3390/healthcare11010111/s1.
