# Supplementary material for: Factors Influencing Food Safety Education Practices among Obstetricians
Source: Healthcare (Basel). 2022 Dec 30;11(1):111. doi: 10.3390/healthcare11010111 (PMC9819058; doi:10.3390/healthcare11010111)
Supplement: Supplementary file 1 [file healthcare-11-00111-s001.zip › healthcare-2050068-supplementary.docx]

**The Questionnaire**

| 1. What is your age? | □22 -29 □30 -39 □40 -49 □ more than 50 | | |
| --- | --- | --- | --- |
| 1. What is your gender? | □Male □Female | | |
| 1. How long have you been in practice? | □0-9 years □10-19 years □≥ 20 years | | |
| **Do you currently discuss with pregnant about?** | | | |
| 1. The risks of toxoplasmosis. | | □Never □Rarely □Sometimes □often □Always | |
| 1. The risks of Listeriosis | | □Never □Rarely □Sometimes □often □Always | |
| 1. The risks of Salmonella | | □Never □Rarely □Sometimes □often □Always | |
| 1. The risks of E. coli | | □Never □Rarely □Sometimes □often □Always | |
| 1. Washing hands appropriately before handling the food? | | □Never □Rarely □Sometimes □often □Always | |
| 1. Washing hands appropriately after touching raw meat, fish, or chicken? | | □Never □Rarely □Sometimes □often □Always | |
| 1. Refrigerator and cook temperatures. | | □Never □Rarely □Sometimes □often □Always | |
| 1. Wearing gloves while gardening | | □Never □Rarely □Sometimes □often □Always | |
| 1. Clean cutting boards, knives, and plates used for raw chicken or meat appropriately before using them for other food? | | □Never □Rarely □Sometimes □often □Always | |
| 1. Clean kitchen items: sponges, cleaning cloths, and kitchen surface as well after use? | | □Never □Rarely □Sometimes □often □Always | |
| 1. Thawing frozen foods. | | □Never □Rarely □Sometimes □often □Always | |
| 1. keep raw food separately from cooked food | | □Never □Rarely □Sometimes □often □Always | |
| 1. Thoroughly reheat all cooked foods or leftovers to boiling before eating? | | □Never □Rarely □Sometimes □often □Always | |
| 1. The risks of eating ready to eat foods served without steaming hot | | □Never □Rarely □Sometimes □often □Always | |
| 1. The risks of eating eggs with runny yolks or foods containing raw egg, such as homemade mayonnaise | | □Never □Rarely □Sometimes □often □Always | |
| 1. The risks of eating pre-prepared salad (Tabola or Fattoush) | | □Never □Rarely □Sometimes □often □Always | |
| 1. The risks of eating undercooked meat or foods containing raw meat, such as raw Koba | | □Never □Rarely □Sometimes □often □Always | |
| 1. The risks of eating unpasteurized milk products. | | □Never □Rarely □Sometimes □often □Always | |
| 1. The risks of eating unwashed fruits and vegetables. | | □Never □Rarely □Sometimes □often □Always | |
| 1. The risks of eating leftovers foods without thoroughly reheating | | □Never □Rarely □Sometimes □often □Always | |
| **Reasons prevent you counseling pregnant women about food safety** | | | |
| 1. I do not have enough time | | | □Strongly disagree □Disagree □ neither agree nor disagree □Agree □ strongly agree |
| 1. I am not a primary source of food safety information | | | □Strongly disagree □Disagree □ neither agree nor disagree □Agree □ strongly agree |
| 1. The clients will not eat the high risk foods anyway. | | | □Strongly disagree □Disagree □ neither agree nor disagree □Agree □ strongly agree |
| 1. I do not have enough knowledge | | | □Strongly disagree □Disagree □ neither agree nor disagree □Agree □ strongly agree |
| 1. Heavy workload and too many patients at the clinics | | | □Strongly disagree □Disagree □ neither agree nor disagree □Agree □ strongly agree |
| 1. Food safety issues are less important to discuss with pregnant women than other topics | | | □Strongly disagree □Disagree □ neither agree nor disagree □Agree □ strongly agree |
| 1. I forget or I need reminders | | | □Strongly disagree □Disagree □ neither agree nor disagree □Agree □ strongly agree |
| 1. Lack of resources and suitable educational tools | | | □Strongly disagree □Disagree □ neither agree nor disagree □Agree □ strongly agree |
| **In your opinion do you think that** | | | |
| 1. Less educated pregnant women need more food safety counseling | | | □Strongly disagree □Disagree □ neither agree nor disagree □Agree □ strongly agree |
| 1. younger pregnant women are less awareness of food safety issues | | | □Strongly disagree □Disagree □ neither agree nor disagree □Agree □ strongly agree |
| 1. It is important to identify food safety education need for pregnant women. | | | □Strongly disagree □Disagree □ neither agree nor disagree □Agree □ strongly agree |
| 1. Provide pregnant women with more food safety education will increase their awareness | | | □Strongly disagree □Disagree □ neither agree nor disagree □Agree □ strongly agree |
| 1. Pregnant women who have more children need less food safety education | | | □Strongly disagree □Disagree □ neither agree nor disagree □Agree □ strongly agree |
| 1. You are interested in taking a continuing education course for health care providers on food safety for high risk groups? | | | □Strongly disagree □Disagree □ neither agree nor disagree □Agree □ strongly agree |
